# The influence of an induced negative emotional state on autobiographical memory coherence

**DOI:** 10.1371/journal.pone.0232495

**Published:** 2020-05-04

**Authors:** Elien Vanderveren, Loes Aerts, Sofie Rousseaux, Patricia Bijttebier, Dirk Hermans

**Affiliations:** 1 Center for the Psychology of Learning and Experimental Psychopathology, KU Leuven, Leuven, Belgium; 2 Undergraduate students clinical and health psychology, KU Leuven, Leuven, Belgium; 3 School Psychology and Development in Context, KU Leuven, Leuven, Belgium; University of St Andrews, UNITED KINGDOM

## Abstract

Individuals who experience difficulty constructing coherent narratives about significant personal experiences generally report less psychological well-being and more depressive symptoms. It remains, however, unclear whether a negative emotional state, one of the core symptoms of depression, causes this impairment in autobiographical memory coherence. The current study aimed to examine the causal relation between mood and memory coherence by means of a mood induction paradigm. A group of 165 students were randomly allocated to one of three mood groups: negative, positive, and neutral. We hypothesized that memory coherence would decrease following a negative mood induction. In addition, working memory capacity was expected to mediate the association between mood and memory coherence. Contrary to predictions, memory coherence increased following a negative mood induction. This increase was likewise observed in the positive mood group, though memory coherence remained consistent in the neutral mood group. This effect of mood on memory coherence was solely observed in female participants and not in the small male subsample. Results provided no support for the hypothesis that working memory capacity functioned as an underlying mechanism. Different theoretical explanations are discussed.

## Introduction

With an estimated 300 million people suffering from major depressive disorder (MDD) worldwide [1], research focusing on increasing insight into core processes of depression that can be targeted to prevent relapse, is of great importance [2,3]. Various features of autobiographical memory have been shown to be compromised in individuals suffering from MDD. For example, recent studies seem to suggest that the ability to narrate autobiographical memories in a coherent manner is impaired in individuals experiencing depressive symptoms [4–6]. However, to this date, it remains unclear whether there is a causal relation between depressive symptoms and this cognitive skill referred to as autobiographical memory coherence.

People differ in the degree to which they are able to create coherent narratives about significant personal experiences. A narrative can be considered coherent if the context in which the events took place is specified and if the events are described in a logical and chronological order. In addition, a coherent narrative contains a clear identifiable theme that is elaborated upon and consists of a highpoint and resolution [7]. The ability to create a coherent narrative is proposed to enhance understanding of the event and to facilitate meaning making [8–10]. It is therefore believed to be an indicator of the emotional processing of those experiences [9,10]. People more adept at constructing coherent narratives report more life satisfaction, self-esteem, feelings of meaning and purpose in life, and experience more positive social relationships [11–13].

In contrast, difficulty with constructing coherent narratives has been shown to be related to depressive symptoms [4–6,11]. There are some indications that especially the ability to construct coherent narratives about negative personal experiences relates to depression [5,6]. Not only memory valence, but gender as well is suggested to moderate the relation between memory coherence and depression. Men are generally less coherent than women [5,6,14,15]. Yet in men, the relation between memory coherence and both psychological well-being and depression seems to be more apparent compared to women. More specifically, previous studies reported that being more adept at constructing coherent memories relates to more well-being and less symptoms of depression, anxiety and stress in men, but not in women [6,14,16]. It remains unclear, however, why men seemingly benefit more from constructing coherent memories than women. One possible explanation could be that, due to gender roles, women typically engage more in emotional disclosure than men, thus doing so in a coherent manner might influence their well-being to a lesser extent [17].

However, the direction of the relation between memory coherence and depression remains unclear. Three pathways seem to be possible. A recent prospective study by Vanderveren, Bijttebier, and Hermans [15] demonstrated that people who are more skilled in creating coherent narratives are better protected against the psychological impact of future negative life events. This seems to suggest that the ability to construct coherent narratives exerts a direct impact on psychological outcomes such as depression. The reverse relationship, however, also seems to be possible, namely that experiencing depressive symptoms directly affects a person's ability to construct coherent narratives. To the best of our knowledge, no prior research has investigated this hypothesis yet. These two pathways are not mutually exclusive though, as the relation could be bidirectional. Memory coherence could have a direct effect on psychological outcomes such as depression and the experience of such symptoms could, reversely, affect the ability to construct coherent narratives. Finally, the relation between memory coherence and depression could also be of an indirect nature, with a third variable underlying their association. The current study will focus on empirically testing the hypothesis that a depressed mood, one of the core symptoms of depression, directly affects narrative coherence and will, additionally, investigate working memory capacity as an underlying mechanism.

A large body of evidence has indicated that a depressed mood can affect several features of autobiographical memories. Depressed individuals recall more negative autobiographical memories and recall them faster compared to positive memories [18,19]. This is consistent with the mood congruency effect that states that memories congruent to the current mood are more likely to be recalled [20]. Moreover, different qualitative aspects of autobiographical memories are impaired in depressed individuals. They recall, for instance, more general and fewer specific autobiographical memories, a consistent observation which is commonly referred to as overgeneral autobiographical memory [21]. Experimental studies implementing a mood induction paradigm have, in addition, demonstrated that depressed individuals recall less vivid and less emotionally intense autobiographical memories [22,23]. In conclusion, autobiographical memory dysfunctions seem to be characteristic of depression.

Besides autobiographical memory impairments, other cognitive functions are distorted in depressed individuals, such as attention, planning, and decision making [24]. Working memory deficiency is thought to explain these cognitive dysfunctions related to depression. Various studies have demonstrated that working memory is impaired in depressed individuals [24,25]. In addition, research has demonstrated that negative affect is associated with poorer working memory performance [26,27]. The Resource Allocation Model is thought to explain this observation. Negative information related to a negative emotional state becomes activated in the working memory, taking up valuable working memory space required to successfully complete other cognitive tasks [28].

Taken together, it seems plausible to propose that a negative mood would impair the ability to create coherent autobiographical narratives. Sufficient executive capacities are required to create coherent narratives [6,7,29]. One has to, for instance, access specific information, organize the events in a chronological order, and formulate a resolution. Recent correlational data show that memory coherence is indeed positively associated with executive functioning [6]. Since negative contents related to the negative emotional state will take up valuable space in the working memory, various executive functions necessary to construct a coherent narrative will be impaired, resulting in less coherent autobiographical narratives.

The primary aim of the present study was to investigate this hypothesis by means of a mood induction paradigm. The study involved three groups of healthy participants, each group receiving one of three mood inductions; negative, positive, or neutral. Memory coherence in the negative mood group was compared with that of the positive and the neutral mood groups. We predicted that memory coherence would decrease following a negative mood induction. No change in memory coherence from pre- to post-mood induction was predicted in the positive and neutral mood groups. Exploratory, the effect of mood on memory coherence will be compared between men and women. In addition, we predicted that working memory capacity would mediate the association between mood and memory coherence. Baseline depressive symptoms and depression history were controlled for, to exclude the possibility that changes in memory coherence would be the result of individual differences in lifetime depression.

## Method

### Participants

The sample consisted of 165 university students, of whom 146 were women (88.5%) and 19 were men (11.5%). Age ranged between 17 and 27 years old (*M* = 19.71, *SD* = 2.02). Participants were randomly allocated to one of three mood induction conditions: neutral condition (*N* = 54), negative condition (*N* = 53), and positive condition (*N* = 58). A total of 30 participants reported a history of depression and, of these, three reported currently experiencing a depressive episode. These participants were not excluded from the analyses and were evenly distributed across conditions (10 participants with a history of depression in each condition). A study with a highly similar design in the field of memory specificity [30] reported an effect size of *d* = .44 for the paired samples t-test in the negative mood condition. Post-hoc power analysis revealed that our adopted sample size of 53 participants in the negative condition resulted in a power of > .80 to detect an effect of that magnitude.

### Instruments

#### Depression

To screen for the presence of depressive symptoms, participants were asked to fill out the Dutch translation of the seven-item depression scale of the *Depression Anxiety Stress Scale-21* (DASS-21) [31,32]. Participants were also asked whether they previously had (i.e., “Have you experienced a depressive episode in the past?”) or currently experienced (i.e., “Are you currently experiencing a depressive episode?”) a depressive episode. Answers to these two questions were combined into a lifetime depression score, which functioned as a proxy measure for depression history. Both this proxy measure as well as current depressive symptoms were used as control variables in the main analyses.

#### Film mood induction

A short film clip was presented to the participants in an attempt to induce a negative, positive, or neutral mood. Following a similar study by Yeung, Dalgleish, Golden, and Schartau [30], three clips were selected. The negative film clip showed the aftermath of an earthquake. It contained pictures of bodies and survivors mourning the loss of loved ones. A clip of a woman winning one million dollars on a game show and celebrating enthusiastically was used to induce a positive mood. Finally, an advertisement of a real estate company was used in the neutral condition. The length of the three film clips ranged between 2 and 3 minutes. Participants were encouraged to empathize with and relate to the persons portrayed in the clips. Meta-analyses have shown several mood induction procedures to be effective, but especially the combination of film clips with the instruction to empathize with the characters [33,34]. The efficacy of the different film clips in inducing the desired mood was piloted in advance (*N* negative group = 13; *N* positive group = 15; *N* neutral group = 13). Condition by Time repeated measures ANOVA revealed a significant interaction effect for both happiness and sadness ratings with a partial eta square of .47 and .51, respectively. Participants from this piloting phase were included in the final sample.

#### Emotion state rating scale

To measure the efficacy of the mood induction, participants were asked before and immediately following the mood induction to rate how intensely they were experiencing 16 different emotions. The scales ranged from *0* (no emotion) to *100* (very intense emotion). The key emotions central to this study were “happy” and “sad”. The other emotions functioned as filler items (scared, angry, disgusted, surprised, guilty, ashamed, courageous, jealous, irritated, regret, proud, relieved, hopeful, and relaxed).

#### Memory coherence

Participants were asked to recall two very negative and two very positive life experiences and to describe each of these experiences using a keyword. Before and after the mood induction, participants received, at random, one negative and one positive keyword to prompt a narrative. Participants were instructed to write about these events in detail and to include not only factual information, but personal evaluations and interpretations as well. To assess memory coherence, two independent raters coded these narratives following the multidimensional coding scheme developed by Reese and colleagues [7]. The characteristics of each dimension (i.e., context, chronology, and theme) are scored on a four-point rating scale ranging from *0* (characteristics are absent) to *3* (characteristics are all present), after which these three sub-scores are summed up to an overall coherence score. This scheme has been established as a reliable method to code for memory coherence [7,13]. Two master students were trained in coding for coherence and inter-rater reliability was computed with a third independent rater. Reliability analysis on 10% of the narratives indicated substantial reliability, as indicated by the following mean Cohen’s Kappa scores across the two master students (κ = .72 for context, κ = .81 for chronology, and κ = .72 for theme).

#### Working memory

To assess working memory capacity, participants completed the GOSPAN [35], which is a Dutch computerized version of the operation span task (OSPAN) [36]. During this task, participants are asked to evaluate the accuracy of simple mathematical equations while trying to remember two-syllable words. At the end of each block, participants are asked to recall these words in the correct order they were presented in. Over the course of the task, the amount of mathematical equations and words increase. Working memory capacity is expressed in the amount of word sequences correctly recalled. The GOSPAN has been established as a reliable and valid procedure to assess working memory capacity [35,37].

### Procedure

Students enrolled at KU Leuven were recruited through different social media platforms to participate in this study. To disguise the actual goal of the study, participants were informed that their skin conductance would be monitored in order to investigate individual differences in stress reactivity during different types of tasks. At the end of the study, participants were informed about this deception. After completion of the study, participants received course credit or €8 as financial compensation. The study was approved by the Social and Societal Ethics Committee of the KU Leuven and was pre-registered on AsPredicted (https://aspredicted.org/9yj2g.pdf).

The course of the experiment was as follows. Participants first completed a questionnaire to screen for the presence of depressive symptoms over the past week and answered two questions concerning depression history. The first mood rating scale was then administered, followed by the selection and brief description in keywords of two very negative and two very positive life experiences. Then two of the four keywords (one negative and one positive) were randomly presented to the participant who was then asked to write a narrative about each keyword. After this first writing assignment, the mood induction took place, which was immediately followed by the administration of the second mood rating scale. Then the participant was asked to create a narrative about the remaining two keywords, after which the GOSPAN was administered. At the end of the study, all participants were shown a happy film clip to restore their mood.

### Data-analysis

SPSS 25.0 was used to analyze the data. Condition by Time repeated measures ANOVAs were performed to assess the effect of the mood induction on memory coherence, after which post-hoc pairwise comparisons were conducted to assess the change in memory coherence from pre- to post-mood induction in each condition. Pearson correlation coefficients were calculated between working memory capacity and changes in mood and memory coherence. Mediation analysis was to be performed using the PROCESS macro developed by Hayes [38].

## Results

### Descriptives

Mean and standard deviation of all variables over conditions are presented in Table 1. Paired samples t-tests revealed that narratives describing a negative experience were more coherent than narratives about a positive experience, *t*(161) = -3.63, *p* < .001, *d* = -.29. In addition, a significant gender difference was observed, with women being more coherent than men, *t*(160) = -3.11, *p* < .01, *d* = -.76. These valence and gender effects were assessed in the sample of narratives constructed before the mood induction. No additional gender differences were observed.

**Table 1:** Descriptive information.

	Min-max	*M*	*SD*
MEMCO_POS pre	0–3	1.41	.68
MEMCO_NEG pre	0–3	1.61	.74
MEMCO_TOT pre	0–3	1.51	.62
MEMCO_POS post	0–3	1.60	.72
MEMCO_NEG post	0–3	1.67	.75
MEMCO_TOT post	0–3	1.63	.63
Depressive symptoms	1–4	1.49	.38
Lifetime depression	0–1	.18	.39
Working memory	0–54	28.86	10.26

MEMCO_POS = coherence of positive memories; MEMCO_NEG = coherence of negative memories; MEMCO_TOT = total memory coherence. The lifetime depression score ranged between 0 and 1, with 0 indicative of no past or current depressive episodes and 1 indicating one or more depressive episodes in the past and/or present.

### Mood induction

The mean emotion ratings before and after the mood induction for the three groups are presented in Table 2. Groups did not differ significantly in their baseline happiness, *F*(2,162) = .88, *p* = .42, *η*_p_^2^ = .01, and sadness ratings, *F*(2,162) = 1.05, *p* = .35, *η*_p_^2^ = .01. There was a significant Time by Condition effect for both the happy, *F*(2,162) = 73.33, *p* < .001, *η*_p_^2^ = .48, and sad ratings, *F*(2,162) = 78.46, *p* < .001, *η*_p_^2^ = .49. Planned paired samples t-tests revealed that reported sadness increased, *t*(52) = -10.48, *p* < .001, *d* = -1.44, whereas happiness decreased, *t*(52) = 10.61, *p* < .001, *d* = 1.46, following the negative mood induction. In the positive condition, the reverse pattern occurred, with sadness ratings decreasing *t*(57) = 5.18, *p* < .001, *d* = .68, and happiness ratings increasing, *t*(57) = -3.81, *p* < .001, *d* = -.50. Happiness ratings remained consistent in the neutral condition, *t*(53) = 0.75, *p* = .45, *d* = .10. Sadness decreased, *t*(53) = 2.21, *p* = .03, *d* = .30, though this difference was small in absolute terms (*M*pre = 21.00, *M*post = 14.17). As to be expected, the film clips did not exclusively affect sadness and happiness ratings, but the whole range of emotions assessed. Condition by Time repeated measures ANOVA revealed partial eta-squares to range between .04 and .40 with an average effect size of .17 for the other emotion ratings. The observed effects on sadness and happiness ratings were, however, the largest.

**Table 2:** Mean and standard deviation (in parentheses) of happiness and sadness ratings before and after mood induction.

	Neutral mood (N = 54)		Positive mood (N = 58)		Negative mood (N = 53)	
	Pre	Post	Pre	Post	Pre	Post
Happiness	62.15 (19.66)	60.43 (20.53)	59.97 (16.22)	68.36 (19.59)	64.28 (15.13)	30.15 (23.21)
Sadness	21.00 (21.59)	14.17 (18.66)	16.22 (16.80)	6.97 (9.66)	17.26 (15.86)	51.91 (20.69)

### Memory coherence

No significant group differences were observed for baseline memory coherence. A Condition (positive, negative, neutral) by Time (pre- to post-mood induction) repeated measures ANOVA with memory coherence as dependent variable revealed no significant interaction effect in the whole sample, *F*(2,157) = 2.11, *p* = .13, *η*_p_^2^ = .03. Exploratory post-hoc analyses, comparing the male versus female sample, revealed a significant interaction effect in women, *F*(2,138) = 3.73, *p* = .03, *η*_p_^2^ = .05, but not in the small subsample of male participants, *F*(2,16), = .80, *p* = .47, *η*_p_^2^ = .09. The interaction in women remained significant after covarying for current depressive symptoms and lifetime depression, *F*(2,136) = 3.39, *p* = .04, *η*_p_^2^ = .05. Table 3 presents women’s mean memory coherence before and after mood induction for each of the three conditions.

**Table 3:** Mean and standard deviation (in parentheses) of women’s memory coherence scores before and after mood induction.

	Neutral mood (N = 49)		Positive mood (N = 52)		Negative mood (N = 45)	
	Pre	Post	Pre	Post	Pre	Post
MEMCO_POS	1.51 (.60)	1.50 (.75)	1.43 (.73)	1.67 (.71)	1.44 (.69)	1.79 (.66)
MEMCO_NEG	1.65 (.66)	1.52 (.69)	1.70 (.73)	1.92 (.76)	1.65 (.70)	1.64 (.73)
MEMCO_TOT	1.58 (.56)	1.51 (.60)	1.58 (.61)	1.79 (.64)	1.53 (.59)	1.71 (.61)

MEMCO_POS = coherence of positive memories; MEMCO_NEG = coherence of negative memories; MEMCO_TOT = total memory coherence.

To assess the change in memory coherence from pre- to post-mood induction in each condition, post-hoc pairwise comparisons were performed. Memory coherence increased in the positive condition, mean difference = -.20, *p* = .01, 95% CI [-.36, -.05], both for positive, mean difference = -.24, *p* = .03, 95% CI [-.45, -.03], and negative narratives, mean difference = -.20, *p* = .03, 95% CI [-.39, .02]. In the negative condition, contrary to expectations, there was an increase in overall memory coherence, mean difference = -.17, *p* = .04, 95% CI [-.33, -.01]. This effect was driven by a significant increase in coherence of positive narratives, mean difference = -.34, *p* < .01, 95% CI [-.57, -.12], whereas the coherence of negative narratives did not change, mean difference = .03, *p* = .76, 95% CI [-.17, .23]. Memory coherence remained stable in the neutral condition, mean difference = .07, *p* = .35, 95% CI [-.08, .23].

### Working memory

Participants with greater working memory capacity were able to create more coherent narratives, *r*(162) = .30, *p* < .00. A one-way ANOVA revealed that working memory capacity did not differ significantly between conditions, *F*(2,162) = 1.80, *p* = .17, *η*_p_^2^ = .02. In addition, working memory was not significantly associated with change in memory coherence from pre- to post-mood induction, *r*(158) = -.05, *p* = .56, nor with change in happiness, *r*(163) = -.12, *p* = 13, or sadness ratings, *r*(163) = .10, *p* = .20. Due to the absence of a significant association between the independent variable (mood) and the mediating variable (working memory), no additional mediation analysis could be performed.

## Discussion

The present study aimed to investigate the causal relation between negative mood and autobiographical memory coherence by means of a mood induction procedure, while taking into account current depressive symptoms and lifetime depression history. Working memory capacity was investigated as a potential underlying mechanism. The following hypotheses were formulated. First, a decrease in memory coherence following a negative mood induction was expected. With regards to the positive and neutral mood groups, no change from pre- to post-mood induction was predicted. Second, it was hypothesized that working memory capacity would mediate the association between memory coherence and mood.

With respect to the first hypothesis, no effect of mood on memory coherence was observed in the whole sample. However, post-hoc analyses in the female subsample did reveal a significant effect of mood on memory coherence, even after taking into account current depressive symptoms and lifetime depression. However, these findings were not in the direction we predicted. Contrary to predictions, a negative mood increased the coherence of narratives describing a positive event, whereas the coherence of negative narratives remained consistent. In addition, memory coherence, both for narratives describing a positive and negative event, increased following a positive mood induction. Memory coherence did not change in the neutral condition. Different theoretical explanations for these findings are provided later on in the discussion. In the small male subsample, the crucial interaction between mood and memory coherence was not statistically significant. As previously mentioned, prior research has indicated that the relation between memory coherence and depression is moderated by gender. Though no conclusions regarding gender differences in the effect of mood on memory coherence can be drawn due to the uneven gender distribution in this study, results suggest this might be a promising future research avenue.

There was no support for the second hypothesis that working memory capacity mediated the association between memory coherence and mood. Working memory did not differ between conditions, nor was it related to changes in memory coherence or changes in happiness and sadness ratings. No mediation analysis could therefore be performed. However, some aspects of the design of this study might have contributed to this null finding. An experimental mood induction procedure only affects mood for a short period of time, usually for about 10 to 20 minutes [39]. Since working memory capacity was assessed only at the end of the study, this assessment could have fallen outside the induction window.

Surprisingly, and contrary to expectations, the coherence of narratives describing a positive experience increased (instead of decreased) following a negative mood induction. Though this is merely a post-hoc explanation, this increase in memory coherence might function as an attempt to repair positive mood. When experiencing a negative mood, healthy individuals will undertake active attempts to repair their mood [40,41]. One way this mood repair can be achieved is by recalling positive autobiographical memories [42,43]. Moreover, the efficacy of this mood repair strategy depends on the way these positive memories are recalled. A concrete, specific processing mood enhances mood repair to the greatest extent [23,44]. Similarly, it is possible that creating coherent narratives, which are more concrete and specific compared to less coherent narratives, would repair a positive mood. Therefore, the observed increase in memory coherence following a negative mood induction might be the result of active efforts to repair positive mood. Nevertheless, additional research is required to further investigate the relation between negative mood and memory coherence. Future studies could, for instance, examine mood after the assessment of memory coherence, to determine whether the increase in coherence actually improved current mood.

The large body of evidence indicating that positive mood improves cognitive performance could shed some light on our finding that memory coherence increased following a positive mood induction [45]. Positive affect has been shown to enhance, among other things, cognitive flexibility, recall, problem solving, and executive functioning [46–48]. It is believed that creating coherent narratives is a cognitive skill that requires recalling specific information related to the experience, organizing the events in a chronological order, and formulating a resolution in order to create meaning [6,7]. So, by facilitating recall, organization, and problem solving, positive affect might subsequently increase memory coherence.

Although the effect of an induced mood on autobiographical memory coherence was solely observed in the female subsample using post-hoc analyses, the observation that a two- to three-minute long film clip affects women’s memory coherence could provide additional theoretical insight regarding memory coherence and its relation to well-being and depression. More specifically, results seem to suggest that memory coherence and the relation between memory coherence and depressive symptoms is dependent on women’s current emotional state. However, given that the present study was the first to experimentally investigate the relation between mood and memory coherence and no statements regarding the effect of mood on memory coherence in men can be made at this point, results should be replicated in a more diverse sample to allow for more comprehensive conclusions. This could then potentially explain some inconsistencies in the literature. Although the negative relation between memory coherence and depressive symptoms has been observed in several studies [4–6,11], some studies have failed to replicate this finding [15,49]. If memory coherence is dependent on current mood, which is not accounted for in the previously mentioned studies, this might contribute to these inconsistencies.

As discussed earlier, three possible pathways regarding the relation between memory coherence and depression are plausible: memory coherence directly affects the experience of depressive symptoms, depressive symptoms directly affect the ability to construct coherent memories, or a third variable underlies the relation between memory coherence and depression. The present study provided no clear support for the second pathway. Although a negative emotional state did have an influence on memory coherence, the effect was opposite to our hypothesis. A longitudinal investigation of the directionality of effects between memory coherence and depression could provide valuable insight into this matter. Similarly, no evidence for working memory as an underlying variable was obtained. Future studies could aim their focus on other potential underlying mechanisms such as rumination or meaning making [6].

Some limitations to the present study should be considered. First, the distribution of gender across the sample was very uneven, with the sample predominantly consisting of female participants. Before general conclusions regarding the relation between negative mood and memory coherence can be drawn, this study should be replicated in more diverse samples. Second, the order in which the narratives were prompted was not counterbalanced, meaning that all participants were asked to narrate about the positive experience first. It could be argued that, given the short period of time mood induction procedures usually affect mood, only the first narrative would be influenced by current emotional state. However, the observation that the coherence of both narratives about positive and negative experiences increased in the positive condition in combination with the fact that the coherence of narratives about positive events did not increase in all conditions seem to suggest that this was not the case. Nonetheless, these are notable limitations that future studies should remedy.
